# PP15 The Appropriateness Of Translation Of Patient-Reported Outcome Measures In Swahili-Speaking Regions Of Sub-Saharan Africa – A Case Study

**DOI:** 10.1017/S0266462325102353

**Published:** 2025-12-29

**Authors:** Magdalena Moshi, Theresia Ambroce Ottaru, Rosemarie Mwaipopo

## Abstract

**Introduction:**

Patient-reported outcome measures (PROMs) are used to quantify health-related quality of life and generate utility data for economic evaluations. Accurate translation and adaptation of PROMs into non-European languages, such as Swahili, is essential for ensuring the intended health information is captured. A case study evaluated the appropriateness of current Swahili PROMs translations and determined whether these captured a person’s health.

**Methods:**

Swahili translations of the common PROMs tool EQ-5D were obtained from EuroQol for all dialects. Native Swahili-speaking experts in health and linguistics reviewed the translations for inconsistencies in key concepts such as “usual activities,” “anxiety/depression,” and “mobility.” The review assessed the adequacy of cultural adaptations, whether translation processes were documented, and the perspective and qualifications of the translators. Qualitative insights were gathered through expert interviews to understand challenges and propose recommendations for achieving cultural and linguistic equivalence in PROMs. The analysis also explored whether the translations addressed sociocultural nuances and aligned with the lived experiences of Swahili-speaking populations.

**Results:**

In total, three versions of the EQ-5D Swahili translation were obtained from EuroQol. The analysis revealed significant inconsistencies in translating concepts like “usual activities,” “anxiety/depression” and “mobility” across Swahili versions. Translation methods were undocumented, and translator qualifications and perspective were not reported. Cultural nuances, such as the sociological and anthropological understanding of anxiety and mobility, were inadequately addressed. For instance, “anxiety/depression” was translated differently across all three translations. Qualitative feedback emphasized the importance of tailoring translations to cultural contexts. The findings informed recommendations for improving translation methods, focusing on transparency, cultural sensitivity, and linguistic accuracy.

**Conclusions:**

There is a need for culturally informed and transparent PROMs translations to ensure accurate health assessments. Engaging local linguistic and medical experts and addressing sociocultural nuances are crucial for improving PROMs validity and reliability. This is particularly important when translating to non-European languages spoken in low-resource settings. When translated correctly, aggregated PROMs data support equitable healthcare decision-making and resource allocation.

